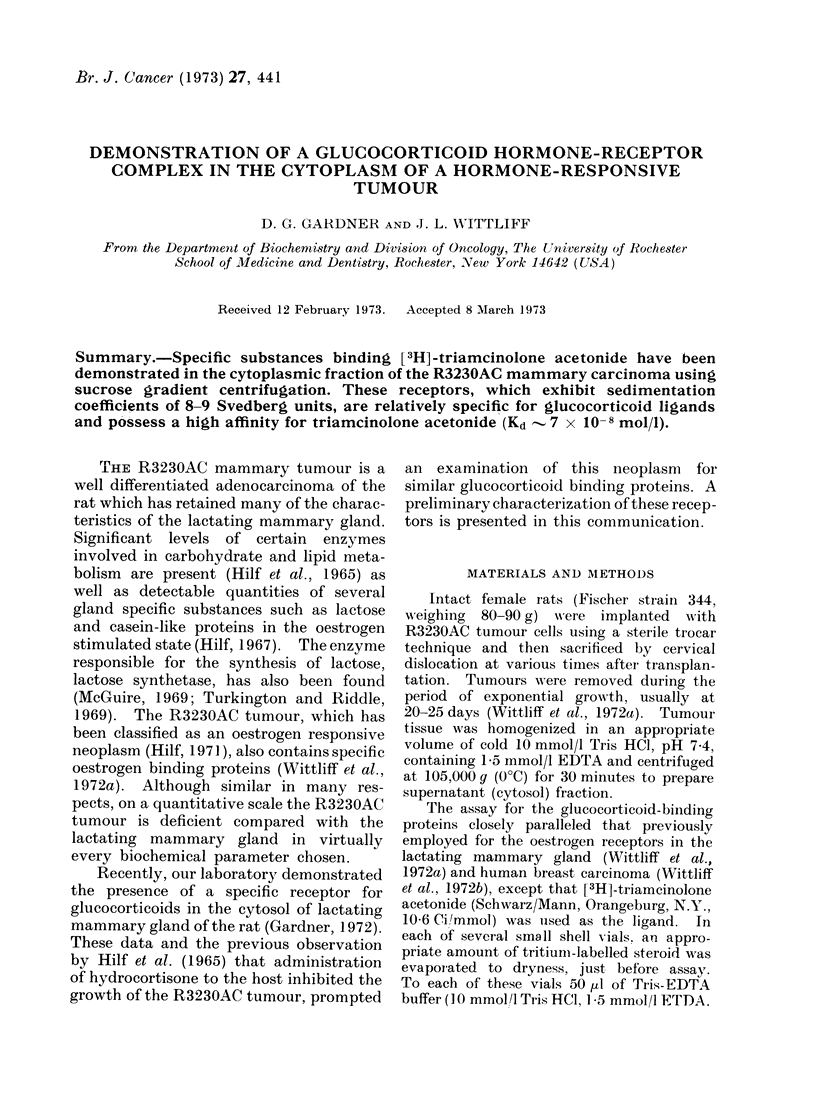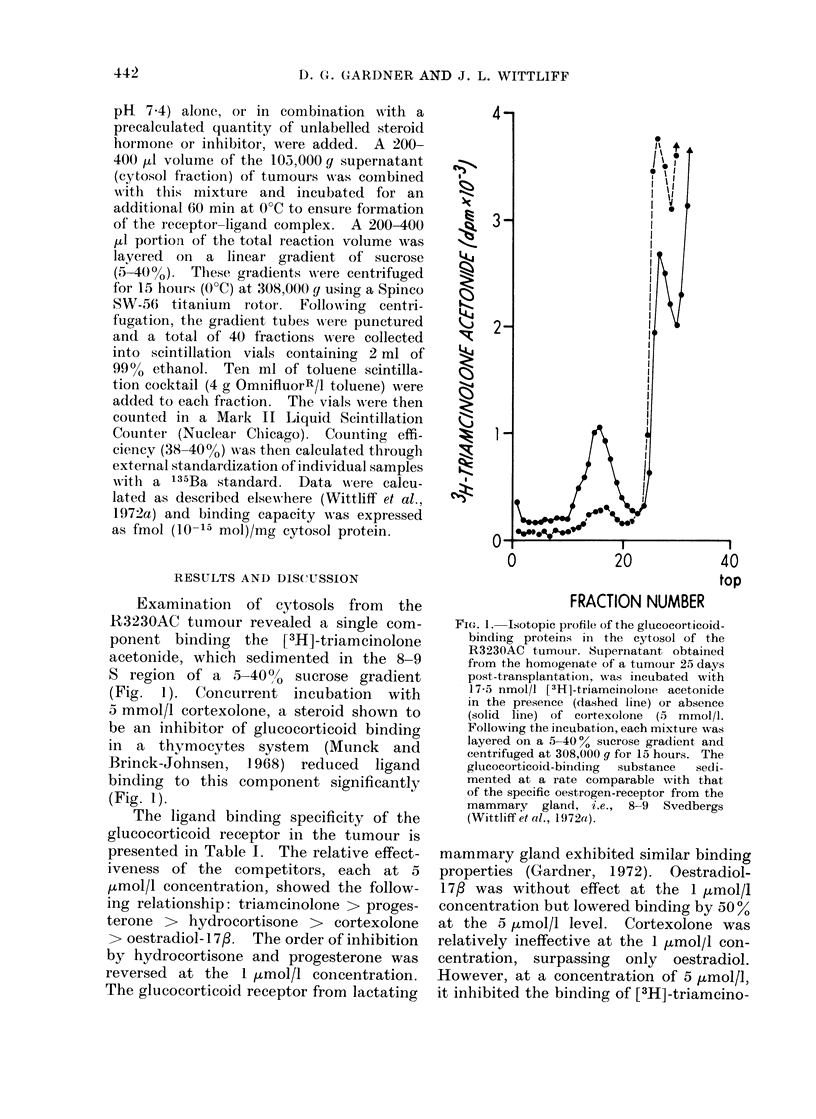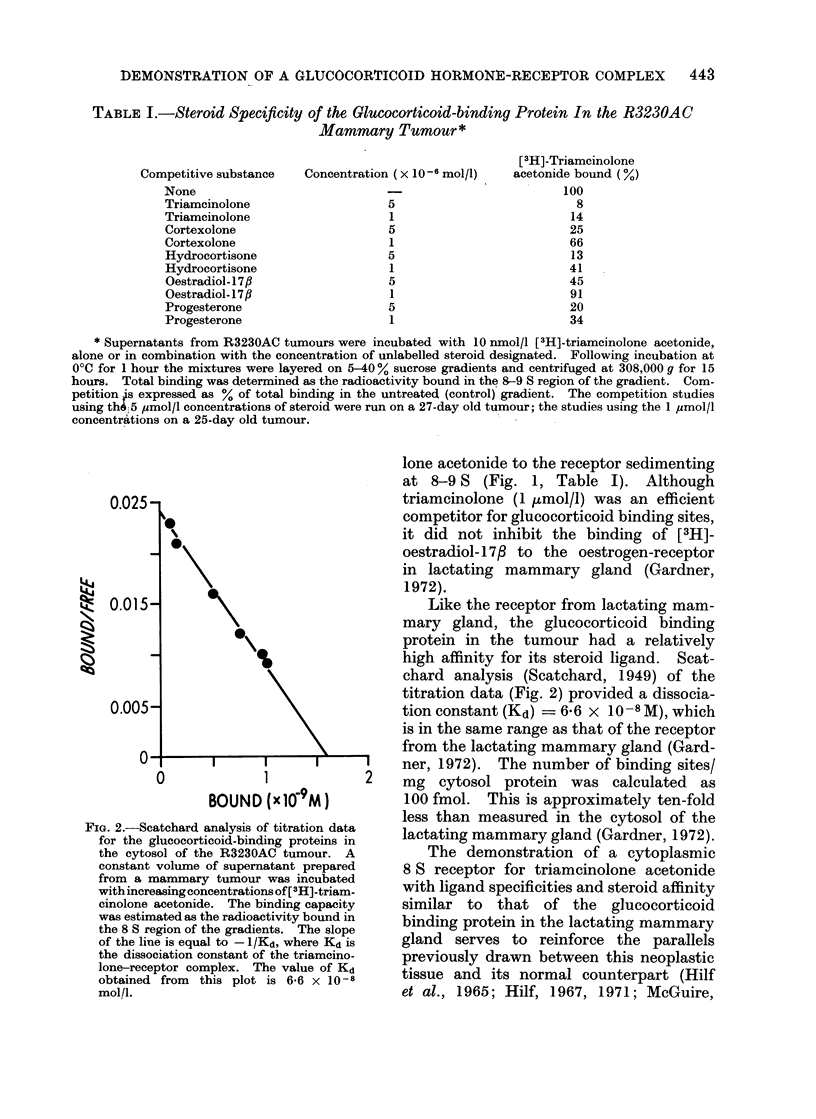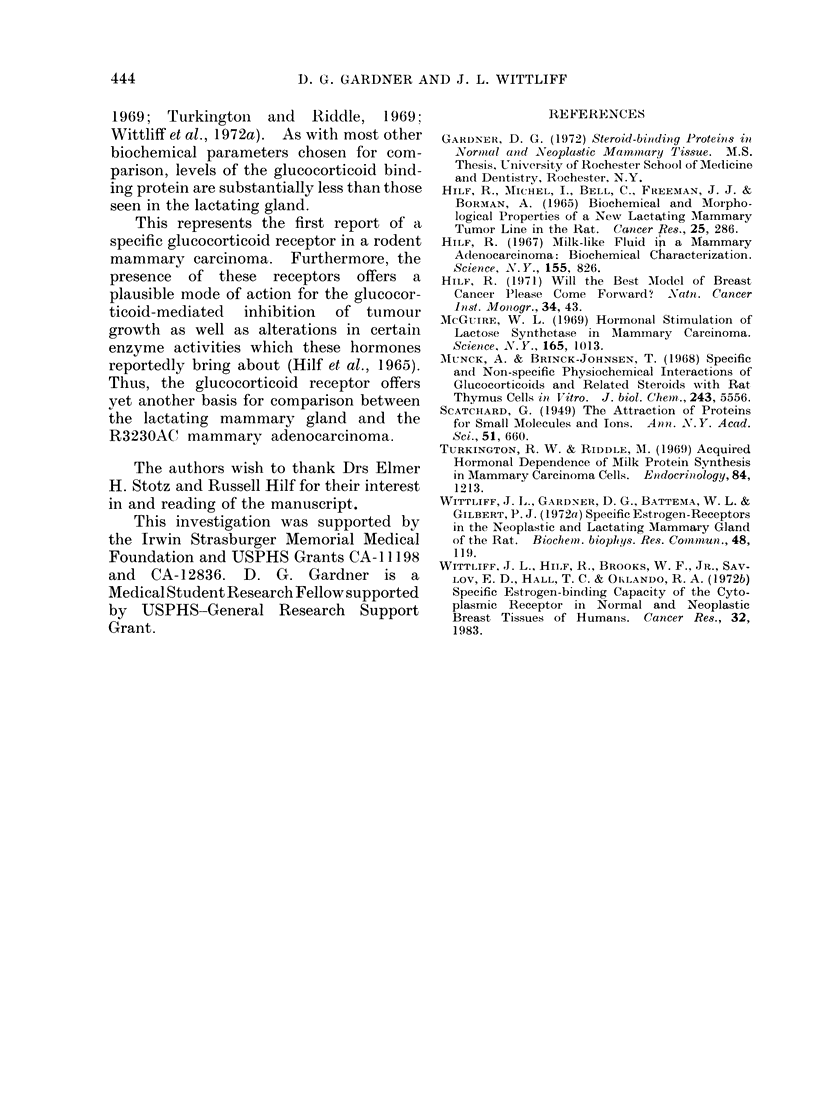# Demonstration of a Glucocorticoid Hormone-receptor Complex in the Cytoplasm of a Hormone-Responsive Tumour

**DOI:** 10.1038/bjc.1973.55

**Published:** 1973-06

**Authors:** D. G. Gardner, J. L. Wittliff

## Abstract

Specific substances binding [^3^H]-triamcinolone acetonide have been demonstrated in the cytoplasmic fraction of the R3230AC mammary carcinoma using sucrose gradient centrifugation. These receptors, which exhibit sedimentation coefficients of 8-9 Svedberg units, are relatively specific for glucocorticoid ligands and possess a high affinity for triamcinolone acetonide (K_d_∼7 × 10^-8^ mol/1).


*Br. J. Cancer* (1973) **27**, 441

### DEMONSTRATION OF A GLUCOCORTICOID HORMONE-RECEPTOR COMPLEX IN THE CYTOPLASM OF A HORMONE-RESPONSIVE TUMOUR

D. G. GARDNER AND J. L. WITTLIFF

*From the Department of Biochemistry and Division of Oncology, The University of Rochester School of Medicine and Dentistry, Rochester, New York 14642 (USA)*

Received 12 February 1973. Accepted 8 March 1973

**Summary.—Specific substances binding [$^3$H]-triamcinolone acetonide have been demonstrated in the cytoplasmic fraction of the R3230AC mammary carcinoma using sucrose gradient centrifugation. These receptors, which exhibit sedimentation coefficients of 8–9 Svedberg units, are relatively specific for glucocorticoid ligands and possess a high affinity for triamcinolone acetonide ($K_d \sim 7 \times 10^{-8}$ mol/l).**

THE R3230AC mammary tumour is a well differentiated adenocarcinoma of the rat which has retained many of the characteristics of the lactating mammary gland. Significant levels of certain enzymes involved in carbohydrate and lipid metabolism are present (Hilf *et al.*, 1965) as well as detectable quantities of several gland specific substances such as lactose and casein-like proteins in the oestrogen stimulated state (Hilf, 1967). The enzyme responsible for the synthesis of lactose, lactose synthetase, has also been found (McGuire, 1969; Turkington and Riddle, 1969). The R3230AC tumour, which has been classified as an oestrogen responsive neoplasm (Hilf, 1971), also contains specific oestrogen binding proteins (Wittliff *et al.*, 1972*a*). Although similar in many respects, on a quantitative scale the R3230AC tumour is deficient compared with the lactating mammary gland in virtually every biochemical parameter chosen.

Recently, our laboratory demonstrated the presence of a specific receptor for glucocorticoids in the cytosol of lactating mammary gland of the rat (Gardner, 1972). These data and the previous observation by Hilf *et al.* (1965) that administration of hydrocortisone to the host inhibited the growth of the R3230AC tumour, prompted an examination of this neoplasm for similar glucocorticoid binding proteins. A preliminary characterization of these receptors is presented in this communication.

### MATERIALS AND METHODS

Intact female rats (Fischer strain 344, weighing 80–90 g) were implanted with R3230AC tumour cells using a sterile trocar technique and then sacrificed by cervical dislocation at various times after transplantation. Tumours were removed during the period of exponential growth, usually at 20–25 days (Wittliff *et al.*, 1972*a*). Tumour tissue was homogenized in an appropriate volume of cold 10 mmol/l Tris HCl, pH 7·4, containing 1·5 mmol/l EDTA and centrifuged at 105,000 *g* (0°C) for 30 minutes to prepare supernatant (cytosol) fraction.

The assay for the glucocorticoid-binding proteins closely paralleled that previously employed for the oestrogen receptors in the lactating mammary gland (Wittliff *et al.*, 1972*a*) and human breast carcinoma (Wittliff *et al.*, 1972*b*), except that [$^3$H]-triamcinolone acetonide (Schwarz/Mann, Orangeburg, N.Y., 10·6 Ci/mmol) was used as the ligand. In each of several small shell vials, an appropriate amount of tritium-labelled steroid was evaporated to dryness, just before assay. To each of these vials 50 $\mu$l of Tris-EDTA buffer (10 mmol/l Tris HCl, 1·5 mmol/l ETDA, pH 7·4) alone, or in combination with a precalculated quantity of unlabelled steroid hormone or inhibitor, were added. A 200–400 μl volume of the 105,000 *g* supernatant (cytosol fraction) of tumours was combined with this mixture and incubated for an additional 60 min at 0°C to ensure formation of the receptor–ligand complex. A 200–400 μl portion of the total reaction volume was layered on a linear gradient of sucrose (5–40%). These gradients were centrifuged for 15 hours (0°C) at 308,000 *g* using a Spinco SW-56 titanium rotor. Following centrifugation, the gradient tubes were punctured and a total of 40 fractions were collected into scintillation vials containing 2 ml of 99% ethanol. Ten ml of toluene scintillation cocktail (4 g Omnifluor$^{R}$/l toluene) were added to each fraction. The vials were then counted in a Mark II Liquid Scintillation Counter (Nuclear Chicago). Counting efficiency (38–40%) was then calculated through external standardization of individual samples with a $^{135}$Ba standard. Data were calculated as described elsewhere (Wittliff *et al.*, 1972*a*) and binding capacity was expressed as fmol ($10^{-15}$ mol)/mg cytosol protein.

Fig. 1.—Isotopic profile of the glucocorticoid-binding proteins in the cytosol of the R3230AC tumour. Supernatant obtained from the homogenate of a tumour 25 days post-transplantation, was incubated with 17·5 nmol/l [$^3$H]-triamcinolone acetonide in the presence (dashed line) or absence (solid line) of cortexolone (5 mmol/l. Following the incubation, each mixture was layered on a 5–40% sucrose gradient and centrifuged at 308,000 *g* for 15 hours. The glucocorticoid-binding substance sedimented at a rate comparable with that of the specific oestrogen-receptor from the mammary gland, *i.e.*, 8–9 Svedbergs (Wittliff *et al.*, 1972*a*).

### RESULTS AND DISCUSSION

Examination of cytosols from the R3230AC tumour revealed a single component binding the [$^3$H]-triamcinolone acetonide, which sedimented in the 8–9 S region of a 5–40% sucrose gradient (Fig. 1). Concurrent incubation with 5 mmol/l cortexolone, a steroid shown to be an inhibitor of glucocorticoid binding in a thymocytes system (Munck and Brinck-Johnsen, 1968) reduced ligand binding to this component significantly (Fig. 1).

The ligand binding specificity of the glucocorticoid receptor in the tumour is presented in Table I. The relative effectiveness of the competitors, each at 5 μmol/l concentration, showed the following relationship: triamcinolone > progesterone > hydrocortisone > cortexolone > oestradiol-17β. The order of inhibition by hydrocortisone and progesterone was reversed at the 1 μmol/l concentration. The glucocorticoid receptor from lactating mammary gland exhibited similar binding properties (Gardner, 1972). Oestradiol-17β was without effect at the 1 μmol/l concentration but lowered binding by 50% at the 5 μmol/l level. Cortexolone was relatively ineffective at the 1 μmol/l concentration, surpassing only oestradiol. However, at a concentration of 5 μmol/l, it inhibited the binding of [$^3$H]-triamcino-

TABLE I.—*Steroid Specificity of the Glucocorticoid-binding Protein In the R3230AC Mammary Tumour**

| Competitive substance | Concentration ( $\times 10^{-6}$ mol/l) | [$^3$H]-Triamcinolone acetonide bound (%) |
|---|---|---|
| None | — | 100 |
| Triamcinolone | 5 | 8 |
| Triamcinolone | 1 | 14 |
| Cortexolone | 5 | 25 |
| Cortexolone | 1 | 66 |
| Hydrocortisone | 5 | 13 |
| Hydrocortisone | 1 | 41 |
| Oestradiol-17β | 5 | 45 |
| Oestradiol-17β | 1 | 91 |
| Progesterone | 5 | 20 |
| Progesterone | 1 | 34 |

* Supernatants from R3230AC tumours were incubated with 10 nmol/l [$^3$H]-triamcinolone acetonide, alone or in combination with the concentration of unlabelled steroid designated. Following incubation at 0°C for 1 hour the mixtures were layered on 5–40% sucrose gradients and centrifuged at 308,000 *g* for 15 hours. Total binding was determined as the radioactivity bound in the 8–9 S region of the gradient. Competition is expressed as % of total binding in the untreated (control) gradient. The competition studies using the 5 μmol/l concentrations of steroid were run on a 27-day old tumour; the studies using the 1 μmol/l concentrations on a 25-day old tumour.

FIG. 2.—Scatchard analysis of titration data for the glucocorticoid-binding proteins in the cytosol of the R3230AC tumour. A constant volume of supernatant prepared from a mammary tumour was incubated with increasing concentrations of [$^3$H]-triamcinolone acetonide. The binding capacity was estimated as the radioactivity bound in the 8 S region of the gradients. The slope of the line is equal to $-1/K_d$, where $K_d$ is the dissociation constant of the triamcinolone–receptor complex. The value of $K_d$ obtained from this plot is $6{\cdot}6 \times 10^{-8}$ mol/l.

lone acetonide to the receptor sedimenting at 8–9 S (Fig. 1, Table I). Although triamcinolone (1 μmol/l) was an efficient competitor for glucocorticoid binding sites, it did not inhibit the binding of [$^3$H]-oestradiol-17β to the oestrogen-receptor in lactating mammary gland (Gardner, 1972).

Like the receptor from lactating mammary gland, the glucocorticoid binding protein in the tumour had a relatively high affinity for its steroid ligand. Scatchard analysis (Scatchard, 1949) of the titration data (Fig. 2) provided a dissociation constant ($K_d$) $= 6{\cdot}6 \times 10^{-8}$ M), which is in the same range as that of the receptor from the lactating mammary gland (Gardner, 1972). The number of binding sites/mg cytosol protein was calculated as 100 fmol. This is approximately ten-fold less than measured in the cytosol of the lactating mammary gland (Gardner, 1972).

The demonstration of a cytoplasmic 8 S receptor for triamcinolone acetonide with ligand specificities and steroid affinity similar to that of the glucocorticoid binding protein in the lactating mammary gland serves to reinforce the parallels previously drawn between this neoplastic tissue and its normal counterpart (Hilf *et al.*, 1965; Hilf, 1967, 1971; McGuire,

1969; Turkington and Riddle, 1969; Wittliff *et al.*, 1972*a*). As with most other biochemical parameters chosen for comparison, levels of the glucocorticoid binding protein are substantially less than those seen in the lactating gland.

This represents the first report of a specific glucocorticoid receptor in a rodent mammary carcinoma. Furthermore, the presence of these receptors offers a plausible mode of action for the glucocorticoid-mediated inhibition of tumour growth as well as alterations in certain enzyme activities which these hormones reportedly bring about (Hilf *et al.*, 1965). Thus, the glucocorticoid receptor offers yet another basis for comparison between the lactating mammary gland and the R3230AC mammary adenocarcinoma.

The authors wish to thank Drs Elmer H. Stotz and Russell Hilf for their interest in and reading of the manuscript.

This investigation was supported by the Irwin Strasburger Memorial Medical Foundation and USPHS Grants CA-11198 and CA-12836. D. G. Gardner is a Medical Student Research Fellow supported by USPHS–General Research Support Grant.

### REFERENCES

GARDNER, D. G. (1972) *Steroid-binding Proteins in Normal and Neoplastic Mammary Tissue.* M.S. Thesis, University of Rochester School of Medicine and Dentistry, Rochester, N.Y.

HILF, R., MICHEL, I., BELL, C., FREEMAN, J. J. & BORMAN, A. (1965) Biochemical and Morphological Properties of a New Lactating Mammary Tumor Line in the Rat. *Cancer Res.*, **25**, 286.

HILF, R. (1967) Milk-like Fluid in a Mammary Adenocarcinoma: Biochemical Characterization. *Science, N.Y.*, **155**, 826.

HILF, R. (1971) Will the Best Model of Breast Cancer Please Come Forward? *Natn. Cancer Inst. Monogr.*, **34**, 43.

MCGUIRE, W. L. (1969) Hormonal Stimulation of Lactose Synthetase in Mammary Carcinoma. *Science, N.Y.*, **165**, 1013.

MUNCK, A. & BRINCK-JOHNSEN, T. (1968) Specific and Non-specific Physiochemical Interactions of Glucocorticoids and Related Steroids with Rat Thymus Cells *in Vitro*. *J. biol. Chem.*, **243**, 5556.

SCATCHARD, G. (1949) The Attraction of Proteins for Small Molecules and Ions. *Ann. N.Y. Acad. Sci.*, **51**, 660.

TURKINGTON, R. W. & RIDDLE, M. (1969) Acquired Hormonal Dependence of Milk Protein Synthesis in Mammary Carcinoma Cells. *Endocrinology*, **84**, 1213.

WITTLIFF, J. L., GARDNER, D. G., BATTEMA, W. L. & GILBERT, P. J. (1972*a*) Specific Estrogen-Receptors in the Neoplastic and Lactating Mammary Gland of the Rat. *Biochem. biophys. Res. Commun.*, **48**, 119.

WITTLIFF, J. L., HILF, R., BROOKS, W. F., JR., SAVLOV, E. D., HALL, T. C. & ORLANDO, R. A. (1972*b*) Specific Estrogen-binding Capacity of the Cytoplasmic Receptor in Normal and Neoplastic Breast Tissues of Humans. *Cancer Res.*, **32**, 1983.